# Is It Ethical to Test Apparently “Healthy” Children for Autosomal Dominant Polycystic Kidney Disease and Risk Medicalizing Thousands?

**DOI:** 10.3389/fped.2017.00291

**Published:** 2018-01-19

**Authors:** Tess Harris

**Affiliations:** ^1^PKD International, Geneva, Switzerland

**Keywords:** autosomal dominant polycystic kidney disease, genetic test, presymptomatic testing, genetic counseling, medicalization, next-generation sequencing

## Introduction

A frequent question asked by parents of children at risk of inheriting autosomal dominant polycystic kidney disease (ADPKD) is: “should my child be tested?” Until recently, most doctors answered “no.” They regarded ADPKD as an “adult” condition and advised families to wait until their children were grown up and not to worry them during childhood. But is this an ethical response today given growing evidence of manifestation in children already diagnosed with ADPKD and the prospect of a treatment (1).

## Background

**Figure 1:**
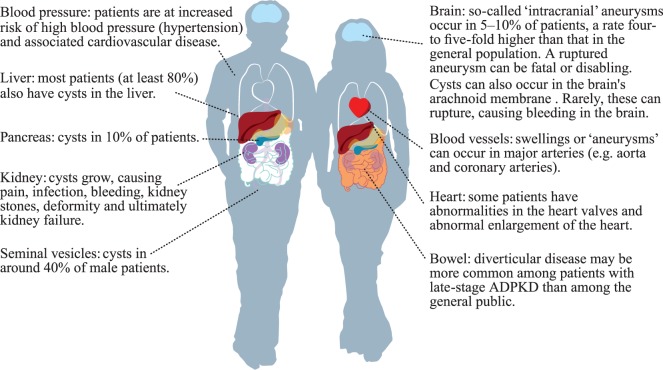
Parts of the body that may be affected by autosomal dominant polycystic kidney disease (ADPKD). The author kindly acknowledges the European ADPKD Forum Report: Translating science into policy to improve ADPKD care in Europe. (2015). Available from: http://www.pkdinternational.org/eaf_adpkd_forum_policy_report_2015/.

Autosomal dominant polycystic kidney disease is the most common inherited renal disease worldwide, affecting 1–2 in 1,000 people (2), an estimated 12.5 million individuals across all ethnic groups. Mutations in three genes (*PKD1, PKD2*, and *GANAB*) cause multiple fluid-filled cysts to develop in both kidneys and other organs. The kidney cysts grow and expand throughout life causing complications, such as chronic and acute pain, infections, hematuria, stones, hernia, and disfiguring abdominal swelling. Over time, the cysts overwhelm and destroy healthy tissue resulting in kidney failure. ADPKD also causes cysts to form in the liver (polycystic liver disease or PLD), with some patients experiencing massive liver growth, infection, and pain. Brain aneurysms are four times as common in ADPKD as the general population and patients are at risk of cardiovascular disease from high blood pressure. Other, rarer manifestations can occur, such as cysts in the pancreas, brain, and seminal vesicles (Figure 1).

Autosomal dominant polycystic kidney disease accounts for one in 10 patients in end-stage renal disease (ESRD) requiring renal replacement therapy (dialysis or kidney transplant) and is a major health-care burden. Across Europe, ESRD in ADPKD is estimated to cost €1.5 billion/year (3). Those with severe PLD may require life-saving liver transplantation due to comorbidities from liver enlargement. Perversely, cystic livers remain functional and patients with PLD can spend years on transplant waiting lists owing to low Model for End-Stage Liver Disease score.

Pre-ESRD, ADPKD patients often experience significant diminution in quality of life. This is due not only to the clinical burden, but also to the impact that this chronic, progressive incurable, genetic disease has on personal and family life. Anxiety, depression, shock, fear of the future, and genetic guilt have been observed in patients (4). Average ESRD age in ADPKD is mid to late 50s (5). Kidney failure at an age when many people are economically productive can result in lost jobs and income—with consequent socio-economic burden.

There are no proven treatments that will stop cyst development. Management is focused on treating the symptoms: anti-hypertensive medication, antibiotics, pain relief, and then dialysis or transplantation. In 2015, the first-ever drug to treat ADPKD in adults was approved by regulators in Japan, Canada, and the EU. In a trial, tolvaptan (a vasopressin V2 receptor antagonist) was shown to help slow ADPKD progression by reducing the rate at which kidneys become enlarged by cysts and helping slow the speed of kidney function decline (6). Tolvaptan does not alter liver cyst growth. In the EU, only adult ADPKD patients with CKD stage 1–3 and evidence of rapidly progressing disease are eligible for this therapy.

## ADPKD in Children

Autosomal dominant polycystic kidney disease is present from conception but symptoms are primarily adult-onset and rare during childhood. Being an autosomal dominant condition, ADPKD carries an inheritance risk of 1 in 2 (50%) from an affected parent. However, *de novo* mutations have been found in 1 in 10–13 people (7). Despite continuous, silent cyst growth, kidney function is typically normal during childhood owing to compensatory hyperfiltration in the nephrons. Many people are not diagnosed until adulthood (even with family histories) when symptoms, such as hypertension, chronic back pain or blood in the urine trigger an investigation for ADPKD. Some patients are diagnosed incidentally, e.g., during scans for kidney stones or pregnancy (unpublished data from PKD Charity UK survey 2015).

Diagnosis is usually by ultrasound. However, cysts in children are not always detected and there is a chance of cyst development in later life. Genetic testing is uncommon owing to cost and genetic complexity. Rarely, genetic testing is used where diagnosis by imaging is inconclusive or to distinguish ADPKD from other cystic kidney diseases such as autosomal recessive polycystic kidney disease and ciliopathies which can “mimic” PKD (8).

There is wide variability in ADPKD progression, even in families with the same mutation. Individuals with ADPKD caused by *PKD1* mutations typically have a more severe progression than those with *PKD2* mutations. ESRD, for instance, can be up to 20 years earlier in patients with *PKD1* mutations (9). Clinical features do overlap, however, and clinicians do not differentiate in practice. Monitoring and treatment are indicated by kidney function progression and comorbidities.

Molecular mechanisms that may explain the variability are starting to be understood. It is suggested that stochastic, epistatic and environmental events may influence ADPKD expression in individuals (10). Advances in next-generation sequencing should provide more knowledge and insights but this technique is not widely available or affordable.

Unless a child has a diagnosis of ADPKD, they probably will not need treatment until later in life. Symptom rarity, perception of ADPKD being an “adult” condition, and lack of licensed treatments for children to slow disease progression, mean that historically doctors and parents felt safe to wait until the child was old enough to decide about testing for themselves.

Presymptomatic testing was discussed at the ADPKD Kidney Disease: Improving Global Outcomes (KDIGO) Consensus Conference (2014). The conference included patients and parents, and at that time there was agreement that minors should not be offered presymptomatic testing (11). Three approaches were proposed for parents considering screening or testing at-risk but undiagnosed children: “screen children as young as possible and disclose the results to the entire family; screen and disclose results only to the parents; do not screen.” Parents would in all cases have the final decision regarding screening.

The KDIGO report’s authors felt that negative outcomes of a positive diagnosis in a child outweighed positives (11). When anyone is diagnosed with ADPKD (or any other potentially life-changing, incurable genetic condition), there are long-term consequences. Career choices may be limited, e.g., patients may be unable to join the armed forces or pursue vigorous contact sports such as rugby or martial arts. Insurance policies for life, critical illness, and private health may be impossible to obtain; other types of insurance will incur higher premiums. A diagnosis has implications for family planning and may negatively influence a patient’s perspective of their future (unpublished data from PKD Charity UK survey 2015).

Undiagnosed at-risk children may appear “healthy” on the outside. However, there is evidence of early symptoms that may be overlooked in the undiagnosed ADPKD population. In a meta-analysis, Marlais et al. estimated that at least one in five children with ADPKD have hypertension (12); in some centers one in three children are affected. Left ventricular hypertrophy is seen. Cyst and kidney growth is evident, sometimes accompanied by pain. Some children have hematuria and urinary tract infections. Early interventions such as anti-hypertensive medication are inexpensive to prescribe and provide long-term positive outcomes with minimal side-effects. Children can be advised about kidney protection during sports.

There is evidence of changing attitudes among clinicians. In a survey, European geneticists, pediatric and adult nephrologists generally agreed that at-risk minors be clinically tested for ADPKD but did not agree about genetic testing (13). The authors called for a consensus, in particular to clarify the sometimes “conflicting information given to families.”

Autosomal dominant polycystic kidney disease was historically called an “adult” condition. The term “adult PKD” is still in use in the SNOMED CT International Codes (14). But it is clearly not just a disease of adulthood, so why are doctors—and parents—reluctant to test children for ADPKD?

## Ethics of Presymptomatic Testing in Children

Although genetic testing is rarely used to diagnose ADPKD, declining costs mean this may become cost-effective and more available. Moreover, the author anticipates there will a rise in parents asking about screening with the potential of access to a licensed therapy (tolvaptan) at 18, as well as growth in clinicians’ awareness of manifestations in children. In the author’s opinion, international recommendations on presymptomatic testing—to encompass both imaging and genetic testing—should be developed as a priority to guide ADPKD screening/testing in children.

It is instructive to examine current recommendations for presymptomatic genetic testing in children and consider customizing them for ADPKD to include imaging. The prevailing view is that children should not be offered genetic tests for inherited conditions that develop in adult life and are currently untreatable. If a test is being considered, parents and doctors need to ask if the test is in the child’s “best interests,” i.e., will it do more harm than good to have a diagnosis or should it wait until the child is sufficiently mature and emotionally competent to decide for themselves?

The European Society of Human Genetics (ESHG) recommends (15) that children (minors) can decide for themselves about genetic testing when they are “well informed, have an adequate understanding of the test and its potential consequences, have the capacity to make this decision, are not exposed to external pressure, and have had appropriate counseling.”

These are laudable recommendations but the author knows from personal family history of ADPKD and 10-year experience moderating a large ADPKD support group (PKD Charity UK) that information and counseling provided to families and individuals undergoing genetic tests are not offered to those considering an ultrasound test. Indeed, many families are given referrals for ultrasound tests without the opportunity to discuss pros and cons. This standard of care is unlikely to change in the short-term as the routine commissioning of genetic services for ADPKD is not expected for many years.

The ESHG recommendations state that presymptomatic testing can be done if preventive interventions can start in childhood. At present, unfortunately, there are no guidelines for the care of children with or at risk of ADPKD and thus no motivation or incentive for physicians to suggest presymptomatic testing (genetic or imaging).

## What are the Medicalization Implications of Presymptomatic Testing of Children at Risk of ADPKD?

It is estimated that 12,000 children (under 18 years) in the UK have ADPKD, of whom about 500 are seen in specialist clinics (unpublished survey of UK nephrologists 2015). The actual undiagnosed population is unknown but presumed to be many thousands. Most children will not have symptoms until adulthood. Many doctors and parents feel that medicalizing children, labeling them with a “disease” while asymptomatic, is inappropriate. Over 90% of parents of these children will also be dealing with the impact of ADPKD on one of them (and possibly other family members). Burdening them with caring for a diagnosed child could add unnecessary anxiety and stress. Moreover, medicalization incurs intervention costs, even if these are limited to hypertensive monitoring and low cost drug prescribing.

Medicalization can have benefits, however (16). Getting a diagnosis can be enabling and empowering. Early anti-hypertensive intervention may reduce cardiovascular risk (and consequent costs) in later life and can help families prepare their children for adulthood. These benefits need to be weighed against the costs to society; there is no immediate solution to this dilemma.

## Opinion

In the author’ opinion, it is ethical to test apparently “healthy” children at risk of ADPKD, but only when there is a specific framework in place for presymptomatic ADPKD testing accompanied by approved international recommendations for follow-up management and long-term care. The framework should set quality standards which ensure: (i) that parents have the opportunity to discuss the implications in advance of referrals for imaging and/or genetic testing; (ii) that testing by ultrasound for ADPKD is considered equivalent to genetic testing and accompanied by access to appropriate counseling; (iii) that the child is involved in the decision-making if they are sufficiently mature and well informed; (iv) that parents are enabled to discuss the diagnostic implications of ADPKD with children and supported by appropriate services and information; and (v) that genetic testing is considered and provided where feasible and cost effective.

## Author Contributions

TH contributed the content of the article, which expresses the personal opinion of TH and not necessarily the official views of the PKD Charity UK and PKD International.

## Conflict of Interest Statement

The author declares that the research was conducted in the absence of any commercial or financial relationships that could be construed as a potential conflict of interest.
